# Does propolis have any effect on rheumatoid arthritis? A review study

**DOI:** 10.1002/fsn3.2684

**Published:** 2022-03-10

**Authors:** Elyas Nattagh‐Eshtivani, Naseh Pahlavani, Golnaz Ranjbar, Jamshid Gholizadeh Navashenaq, Ammar Salehi‐Sahlabadi, Trias Mahmudiono, Mohammed Nader Shalaby, Mohammadhassan Jokar, Mohsen Nematy, Hanieh Barghchi, Shahrzad Havakhah, Mona Maddahi, Mohammad Rashidmayvan, Maryam Khosravi

**Affiliations:** ^1^ 37552 Student Research Committee Mashhad University of Medical Sciences Mashhad Iran; ^2^ 37552 Department of Nutrition School of Medicine Mashhad University of Medical Sciences Mashhad Iran; ^3^ Health Sciences Research Center Torbat Heydariyeh University of Medical Sciences Torbat Heydariyeh Iran; ^4^ Children Growth and Development Research Center Research Institute for Prevention of Non‐Communicable Disease Qazvin University of Medical Sciences Qazvin Iran; ^5^ 394237 Noncommunicable Diseases Research Center Bam University of Medical Sciences Bam Iran; ^6^ 556492 Student Research Committee Department of Clinical Nutrition and Dietetics School of Nutrition and Food Technology Shahid Beheshti University of Medical Sciences Tehran Iran; ^7^ 148005 Departmentof Nutrition Faculty of Public Health Universitas Airlangga Airlangga Indonesia; ^8^ 68831 Biological Sciences and Sports Health Department Faculty of Physical Education Suez Canal University Ismailia Egypt; ^9^ 37552 Rheumatic Diseases Research Center School of Medicine Mashhad University of Medical Sciences Mashhad Iran; ^10^ 37552 Metabolic Syndrome Research Center Mashhad University of Medical Sciences Mashhad Iran; ^11^ Addiction and Behavioral Sciences Research Center North Khorasan University of Medical Sciences Bojnurd Iran

**Keywords:** inflammation, oxidative stress, propolis, rheumatoid arthritis

## Abstract

Rheumatoid arthritis (RA) is a chronic autoimmune disease in which inflammation and oxidative stress play a key role in its pathophysiology. Complementary therapies along with medications may be effective in the control of RA. Propolis is a natural substance extracted from beehives, which have confirmed anti‐inflammatory and antioxidant effects. The present study aimed to review the possible effects of propolis on inflammation, oxidative stress, and lipid profile in patients with RA. English articles in online databases such as PubMed‑Medline, AMED, Google Scholar, EMBASE, Scopus, and Web of Science databases were searched. Pieces of evidence show that supplementation with propolis may have therapeutic effects on RA patients. Due to increased inflammation and oxidative stress in the affected joints of RA patients, propolis could inhibit the inflammatory cascades by inhibiting the nuclear factor kappa B pathway and reducing reactive oxygen species, malondialdehyde, and interleukin‐17 by increasing some antioxidants. Therefore, inflammation and pain reduce, helping improve and control RA in patients. Further investigations are required with larger sample sizes and different doses of propolis to demonstrate the definite effects of propolis on various aspects of RA.

## Graphical abstract

Rheumatoid arthritis (RA) is an autoimmune and systemic disorder, and cytokines and inflammatory responses play a key role in its pathogenesis. Antioxidant supplements may help reduce the symptoms and improve the quality of life in RA patients. Propolis has numerous antioxidant and polyphenolic properties and may exert beneficial effects on the oxidative stress biomarkers and inflammation process.

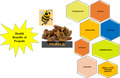

## 1 INTRODUCTION

Rheumatoid arthritis (RA) is a heterogeneous autoimmune and systemic disorder in which cytokines and inflammatory responses play a key role in its pathogenesis (Lubberts & van den Berg, 2013; Nattagh‐Eshtivani et al., 2021). Chronic inflammation starts in the synovial membrane and develops into subsequent lesions in the joint cartilage (Lubberts & van den Berg, 2013). The prevalence of RA is estimated at 0.5%–1.0% in the adult population worldwide (Vaghef‐Mehrabany et al., 2016). The risk of mortality is higher in patients with RA than in the general population (Helli et al., 2016). Evidence suggests that the higher mortality rate of RA patients is due to the increased cardiovascular risk (Myasoedova & Gabriel, 2010).

Despite the extensive research that has unveiled some of the contributing factors to the initiation and development of RA, the exact etiology of the disease remains unknown (Tobón et al., 2010). Oxidative stress and inflammation may be significantly involved in the physiopathology of RA, and evidence attests to the increased level of oxidative stress biomarkers and decreased blood antioxidants in patients with RA (Filippin et al., 2008; Kalpakcioglu & Şenel, 2008; Kamanlı et al., 2004; Taysi et al., 2002). Furthermore, it is proposed that reactive oxygen species (ROS) could cause inflammatory responses in RA by activating nuclear factor kappa B (NF‐κB) (Filippin et al., 2008). Therefore, using antioxidant supplements may help reduce the symptoms and improve the quality of life in RA patients.

The treatment of RA patients with nonsteroidal anti‐inflammatory drugs, glucocorticoids, and disease‐modifying antirheumatic drugs could ameliorate the symptoms, although the patients may experience complications such as osteoporosis, diabetes mellitus, and weight gain; these treatments are also rather expensive (Gautam & Jachak, 2009; Mousa et al., 2021; O'Dell, 2004). Therefore, complementary treatments have attracted the attention of researchers to reduce the complications and costs of RA treatment. Studies regarding herbal medicines have confirmed the beneficial effects of medicinal plants on the prevention and management of chronic diseases such as RA (Ernst, 2010; Kaur et al., 2012; Sarker et al., 2020).

Propolis (bee glue) is a resinous substance that honeybees collect from various plants and mix with wax and other secretions for the construction and protection of beehives (Barlak et al., 2011; Cardoso et al., 2011; Pascoal et al., 2014; Sforcin & Bankova, 2011). Propolis contains more than 300 chemical compounds, such as flavonoids (flavones, flavanones, flavonols, and dihydroflavonols), fatty acids, phenolic acids, aliphatic and aromatic acids, steroids, amino acids, polyphenolic acids, alcohols, terpenes, sugars, and esters (Bankova, 2005; Barlak et al., 2011; Nattagh‐Eshtivani et al., 2021). The numerous biological and pharmacological properties of propolis have also been confirmed, including antimicrobial (Scazzocchio et al., 2006; Uzel et al., 2005), antiviral (Kujumgiev et al., 1999), antifungal (Kujumgiev et al., 1999), free radical‐scavenging (Ichikawa et al., 2002; Scheller et al., 1990), anti‐inflammatory (Boufadi et al., 2021), hepatoprotective, anticancer, antioxidant (Russo et al., 2002), antineurodegenerative, and immune system‐stimulating properties (Farooqui & Farooqui, 2012; Sforcin, 2007). Moreover, propolis has been reported to positively influence cartilage, bones, and dental pulp regeneration (Ramos & Miranda, 2007). The aim of this study was to review the previous publications on the anti‐inflammatory, antioxidant, and cardioprotective effects and also its possible mechanisms related to propolis supplementation in RA disease.

## 2 METHODS

A systematic search was carried out in the PubMed‑Medline, AMED, Google Scholar, EMBASE, Scopus, and Web of Science databases by two independent reviewers (M. M. and H. B.) to identify the relevant articles. The key terms, including “propolis,” in combination with “inflammation,” “oxidative stress,” “antioxidant,” “anti‐inflammatory,” “dyslipidemia,” “lipid profile,” “cardiovascular disease,” “heart disease,” “atherosclerosis,” and “inflammatory markers,” were used for an electronic search strategy. After checking the titles and abstracts of the resulting articles, all the relevant studies published only in English were reviewed subsequently; the eligible animal and clinical trials studies were selected, and duplicate citations were then removed.

### 2.1 The effects of propolis supplementation on inflammation in RA

Ample evidence suggests that systemic inflammation plays a key role in the development and progression of several chronic diseases, including RA (Balkarli et al., 2016; Laveti et al., 2013). As such, diminishing inflammation may be associated with the reduced risk of RA. In addition, environmental factors have been shown to induce immune cell responses, causing the immune system to release large amounts of pro‐inflammatory cytokines (Fox, 2005). Among these cytokines, tumor necrosis factor‐alpha (TNF‐α), interleukin‐1 beta (IL‐1β), and interleukin‐6 (IL‐6) could cause joint degradation by inducing inflammation and synovial cell activation (Furst et al., 2003; Weinblatt et al., 1999; Yaykasli, 2013).

**FIGURE 1:**
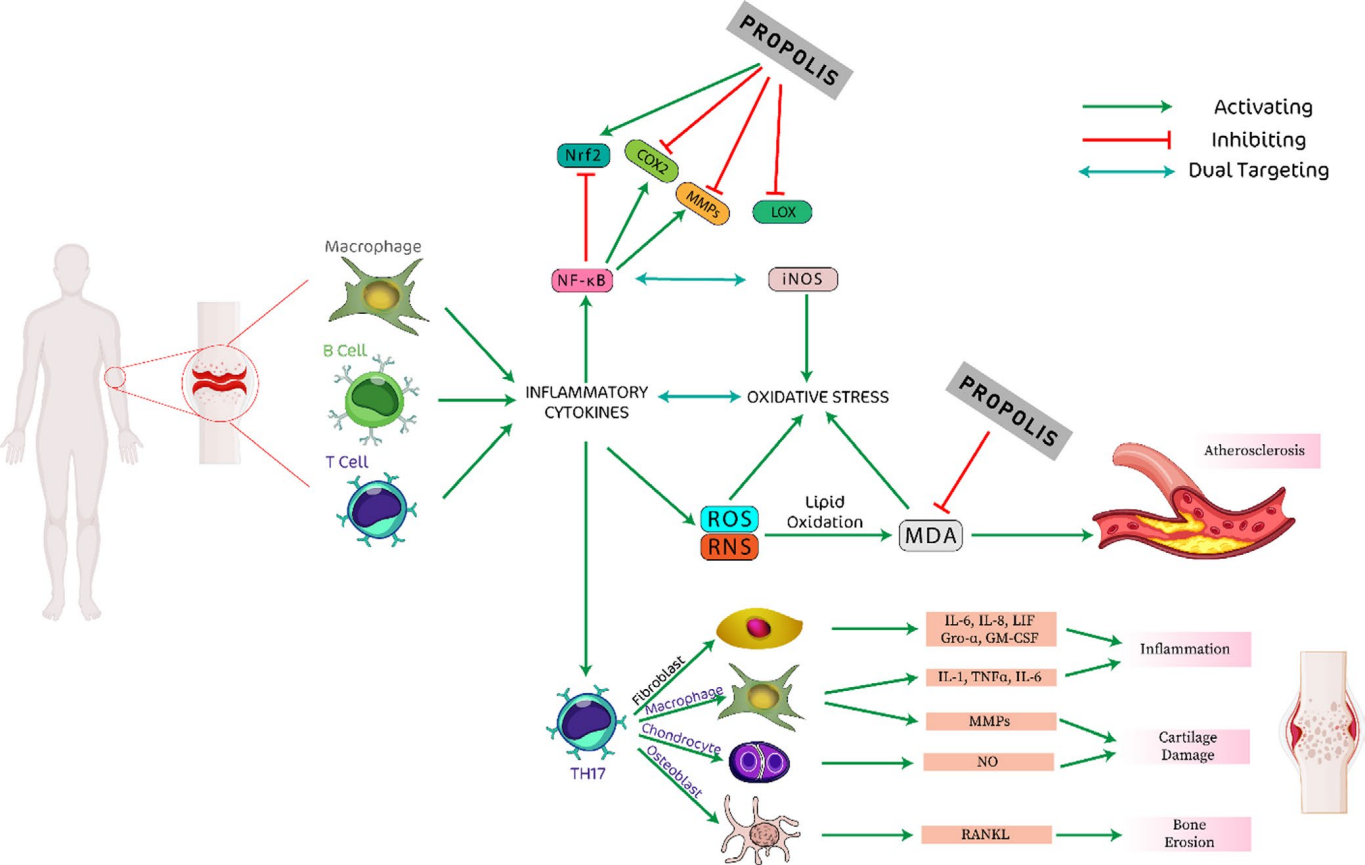
Hypothetical mechanism of effects of propolis on reduction of inflammation, oxidative stress, and atherosclerosis

Inflammatory cascades are responsible for the overexpression of TNF‐α. This cytokine leads to synovitis, articular damage, and overproduction of other cytokines, particularly IL‐6, which increases inflammation and joint degeneration (Scott et al., 2010). Interleukin‐17 (IL‐17) is another pro‐inflammatory cytokine, the catabolic effects of which are mediated by its ability to stimulate cartilage and bone degradation (Figure 1) (Koenders et al., 2005; Van Den Berg & Miossec, 2009). Therefore, these cytokines are often targeted in the treatment of RA patients, and researchers have attempted to effectively prevent and manage the inflammatory cascade by using dietary supplements, which have fewer side effects and are cost‐efficient.

Propolis as a complementary medicine has been used in the treatment of various diseases (Farooqui & Farooqui, 2012; Fukuda et al., 2015; Hu et al., 2005; Santos, 2012), and investigations in this regard have confirmed that propolis and its flavones could cause reduction in inflammation (Afsharpour et al., 2017; De Almeida & Menezes, 2002; Jalali et al., 2020). Furthermore, several studies have been conducted on animal models (Table 1). Fang et al. (2013) reported that 160 mg/kg/day of the ethanol extract of propolis (EEP) could significantly decrease IL‐6 in mice after 14 weeks of treatment. In another study, Corrêa et al. observed that 100 mg/kg/day of Brazilian red propolis reduced the IL‐6 and TNF‐α levels in mice after 9 days of administration (Corrêa et al., 2017). The findings of Kismet et al. also demonstrated that the intraperitoneal daily dosage of propolis (200 mg/kg) could significantly decrease TNF‐α and IL‐6 in rats with nonalcoholic fatty liver disease after 2 weeks of treatment (Kismet et al., 2017). In another study, the administration of propolis by gavage (500 mg/kg/day) for 4 days has shown reduction in the intraperitoneal permeability of mice by lowering the effects of inflammatory factors (Lima et al., 2014).

**TABLE 1:** Summary of animal studies on anti‐inflammatory effects of propolis

First author (year)	Country	Subjects	Administered dose of propolis	Duration (day)	Outcomes	Reference
Hu et al. (2005)	China	Mice	1 ml/100 g	–	↓ IL‐6	Hu et al. (2005)
Machado et al. (2012)	Brazil	Mice	5 mg/kg/day	6	↓ IL‐6 and TNF‐α levels	Machado et al. (2012)
Fang et al (2013)	China	Mice	160 mg/kg/day	98	↓ IL‐6	Fang et al. (2013)
Hemieda et al. (2015)	Egypt	Rat	50/100 mg/kg/day	42	↓ CRP, TGF‐β	Hemieda et al. (2015)
Elissa et al. (2015)	Egypt	Rat	0.6 g/kg/day	21	↓ TNF‐α	Elissa et al. (2015)
Corrêa et al. (2017)	Brazil	Mice	100 mg/kg/day	9	↓ IL‐6 and TNF‐α levels	Corrêa et al. (2017)
Wang et al. (2018)	China	Rat	300 mg/kg/day	7	Colonic inflammatory markers IL‐1β, IL‐6 suppressed by propolis	Wang et al. (2018)
Kismet et al. (2017)	Turkey	Rat	200 mg/kg	14	↓ TNF‐α and IL‐6 levels	Kismet et al. (2017)
El Rabey et al. (2017)	Saudi Arabia	Rat	20% w/w	28	↓ IL‐6	El Rabey et al. (2017)
Chen et al. (2018)	Taiwan	Rat	919.5 mg/kg/day	56	↓ TNF‐α, IL‐1β, and IL‐6	Chen et al. (2018)

Abbreviations: ↑, increase; ↓, decrease; ↔, no effect; CRP, C‐reactive protein; IL, interleukin; TGF‐β, transforming growth factor β; TNF‐α, tumor necrosis factor‐alpha.

According to the study by Chen et al., propolis gavage (919.5 mg/kg/day) could decrease serum TNF‐α, IL‐1β, and IL‐6, whereas a lower dose (183.9 mg/kg/day) induced moderate responses in terms of TNF‐α and IL‐1β levels (Chen et al., 2018). Furthermore, Cheung K. W. et al. reported that Brazilian propolis and its components (artepillin C) inhibited IL‐17 production in human CD4 T cells (Cheung et al., 2011). Therefore, it could be concluded that propolis has antiarthritic effects as T‐helper 17 cells, which are involved in the pathogenesis of RA (Iwakura & Ishigame, 2006; Steinman, 2007). Therefore, it is suggested that propolis supplementation in patients with RA could control the disease by decreasing the inflammatory cascade and the secretion of pro‐inflammatory indices. Tables 1 and 2 summarize the animal studies and clinical trials regarding the anti‐inflammatory effects of propolis, respectively.

**TABLE 2:** Summary of clinical trials on anti‐inflammatory effects of propolis

First author (year)	Country	Sample size (T/C)	Subjects	Type and dose of propolis	Duration (week)	Outcomes	Reference
Zhao et al. (2016)	China	32/33	T_2_DM	900 mg/day	18	↓ TNF‐α ↑ IL‐6	Zhao et al. (2016)
Khayyal et al. (2002)	Egypt	22/24	Patients with mild‐to‐moderate asthma	2 ml/day	8	↓ TNF‐α, IL‐6, and IL‐8 ↑ IL‐10	Khayyal et al. (2002)
Fukuda et al. (2015)	Japan	41/39	T_2_DM	226.8 mg/day	8	↔ CRP, ↔ TNF‐α and IL‐6	Fukuda et al. (2015)
Gao et al. (2018)	China	25/30	T_2_DM	900 mg/day	18	↑ IL‐6	Gao et al. (2018)
Mujica et al. (2017)	Chile	35/32	Healthy subjects	–	12	↔ CRP	Mujica et al. (2017)
Afsharpour et al. (2017)	Iran	30/30	T_2_DM	1500 mg/day	8	↓ CRP and TNF‐α	Afsharpour et al. (2017)
Zhu et al. (2018)	China	30/30	Elderly subjects	66 mg/day	96	↓ IL‐6	Zhu et al. (2018)
Zakerkish et al. (2019)	Iran	50/44	T_2_DM	1000 mg/day	12	↓ CRP and TNF‐α ↔ IL‐6	Zakerkish et al. (2019)
Gholaminejad et al. (2019)	Iran	29/28	Men with asthenozoospermia	1500 mg/day	10	↓ CRP and TNF‐α	Gholaminejad et al. (2019)
Darvishi et al. (2020)	Iran	26/24	Patients with breast cancer	500 mg/day	12	↔ TNF‐α	Darvishi et al. (2020)
Soleimani et al. (2021)	Iran	24/25	Healthy subjects	900 mg/day	4	↓ IL 6	Soleimani et al. (2021)

Abbreviations: ↑, Increase; ↓, decrease; ↔, no effect; C, control; CRP, C‐reactive protein; IL, interleukin; T, treatment; T_2_DM, type II diabetes mellitus; TNF‐α, tumor necrosis factor‐alpha.

### 2.2 Anti‐inflammatory mechanism of propolis

During the inflammation process, macrophages activate and release pro‐inflammatory cytokines such as TNF‐α, IL‐1, and IL‐6. These activated macrophages induce the translocation of NF‐κB. NF‐κB activation plays a pivotal role in the production and stimulation of various cytokines and inflammatory mediators (TNF‐α, IL‐1, IL‐2, IL‐6, and IL‐8) while also participating in the regulation of inflammation (Baeuerle, 1991; Surh et al., 2001). Furthermore, NF‐κB is critically involved in modulating the survival, differentiation, and activation of immune cells (Liu et al., 2017). The NF‐κB signaling pathway also partakes in the production of nitric oxide (NO) by stimulating inducible nitric oxide synthase (iNOS), which is an inflammatory mediator (Pahlavani et al., 2019; Xie et al., 1994).

Propolis has potent anti‐inflammatory activities (Ying‐Hua et al., 2012). It reduces the gene expression of iNOS, the cytokines mediated by NF‐κB activation, and the immune response in T cells (Banskota et al., 2001; Paulino et al., 2008). Also as demonstrated in previous research, propolis components could have directly regulated the basic immune cell functions (Wolska et al., 2019). For example, in lipopolysaccharide‐stimulated RAW264.7 macrophages, neovestitol, an isoflavonoid derived from propolis, showed an immunological modulatory impact by inhibiting NO production and lowering pro‐inflammatory cytokine levels (Bueno‐Silva et al., 2017). Propolis extracts and propolis compounds (caffeic acid, phenethyl ester, quercetin, and hesperidin) could suppress DNA synthesis and the production of inflammatory cytokines (IL‐1, IL‐12, IL‐2, and IL‐4) in Th1‐ and Th2‐type T cells while enhancing the production of transforming growth factor‐β1 (TGF‐β1) (Ansorge et al., 2003). Furthermore, the suppression of macrophage activation and differentiation has been proposed as one of the possible mechanisms causing propolis’ anti‐inflammatory and immunological benefits (Araujo et al., 2012).

Caffeic acid phenethyl ester (CAPE) is an important component of propolis with anti‐inflammatory properties (Tolba et al., 2013). Evidence suggests that CAPE is a potent modulator of arachidonic acid (AA) that blocks the release of AA from the cell membrane, thereby suppressing the gene expression of lipoxygenase and cyclooxygenase (COX) enzymes (Mirzoeva & Calder, 1996). According to various investigations, CAPE is a dominant and selective inhibitor of NF‐κB activation; CAPE has been shown to inhibit NF‐κB activation precisely and completely by a wide range of inflammatory stimuli, including TNF‐α and H_2_O_2_ (Ramos & Miranda, 2007).

Propolis also prevents the production of leukotriene and prostaglandin. Propolis flavonoids may be responsible for their effects on the COX enzyme, which has been reported to suppress prostaglandin‐endoperoxide synthase (Mirzoeva & Calder, 1996). In this regard, Woo et al. examined the effects of chrysin on the expression of COX‐2, reporting that chrysin could significantly suppress the expression of COX‐2 protein and mRNA (Woo et al., 2005). In an in vitro study by Kao et al., the anti‐inflammatory effects of artepillin C were investigated on mice, and the obtained results indicated that artepillin C inhibited prostaglandin E2 synthesis and NO production while also reducing NF‐κB activity in mice (Kao et al., 2010).

Notably, the anti‐inflammatory effects of quercetin have been attributed to the downregulation of the extracellular signal‐regulated kinase, p38, Akt, Janus kinase‐1, tyrosine kinase 2 (TYK2), signal transducer, and NF‐κB activator. This compound has also been shown to scavenge free radicals (Kao et al., 2010). Furthermore, a large number of other flavonoids such as apigenin, galangin, and pinocembrin are found in propolis. Zhang et al. reported that apigenin reduced the mRNA levels of IL‐1, IL‐6, and TNF‐α in human THP‐1‐derived macrophages (Zhang et al., 2014). In addition, pinocembrin significantly reduced the levels of these pro‐inflammatory cytokines in RAW 264.7 macrophage cells, whereas it significantly increased the levels of IL‐10 (Soromou et al., 2012). Galangin significantly lowered the levels of IL‐6 and TNF‐α cytokines in the same RAW 264.7 cell line (Lee et al., 2018). In vivo, oral administration of propolis in C57BL/6 mice for 14 days inhibited spleen cell production of IL‐1, IL‐6, IL‐2, IL‐10, and IFN‐γ (Missima et al., 2010). Moreover, an ethanolic extract of Brazilian propolis inhibited the expression of IL‐17 in mice with collagen‐induced arthritis (Tanaka et al., 2012). Another study found that the anti‐inflammatory activity of Brazilian green propolis in stimulated J774A.1 macrophages is mediated through the inhibition of NO and pro‐inflammatory cytokines such as TNF‐α, IL‐1, and IL‐6 (Szliszka et al., 2013). As a result, propolis and its ingredients might exert potential natural anti‐inflammatory agents that work by modifying immune responses.

### 2.3 Effects of propolis in relation to oxidative stress in RA

Although the exact etiology of RA remains unknown, several studies have confirmed the role of ROS in the pathophysiology of the disease (Bauerova & Bezek, 2000). ROS are naturally produced during aerobic metabolism, and the cells are protected against ROS by the antioxidant defense system (Roy et al., 2017). When ROS production exceeds the capacity of the antioxidant system, oxidative stress occurs and causes metabolic dysfunction and extensive damage to fats, proteins, and DNA. Ultimately, the free radicals produced from oxygen metabolism destroy the antioxidant system (Tao et al., 2018).

In RA, the activation of neutrophils and macrophages (main cells of inflammatory synovial fluid) increases the production of ROS, which are important mediators of tissue damage in arthritis (Kamanlı et al., 2004; Oztürk et al., 1999). On the contrary, malondialdehyde (MDA) is the main indicator of lipid peroxidation, which increases in synovial fluid of RA patients. Previous findings have suggested that MDA increases in the serum, plasma, and synovial fluid in RA under normal conditions (Das et al., 2020). Studies have also shown that serum antioxidants are lower in RA patients than in healthy individuals, and the activity of antioxidant enzymes such as glutathione peroxidase (GPX), superoxide dismutase (SOD), and catalase (CAT) is altered in the serum of these patients. However, contradictory results have been proposed in this regard (Akyol et al., 2001; Kiziltunc et al., 1998; Sarban et al., 2005).

In addition to acting as a protective mechanism against ROS, antioxidants could suppress the expression of the cytokines and collagenase induced by TNF‐α, which is also a protective mechanism against arthritis (Halliwell et al., 1988; Sato et al., 1996). It is hypothesized that natural compounds with antioxidant properties may exert protective effects against RA (Bae et al., 2003; Wang et al., 2019). Propolis is a natural compound that is expected to be effective in reducing oxidative stress levels (Abass et al., 2017; Mujica et al., 2017; Pahlavani et al., 2020). Several studies have demonstrated that propolis could also decrease oxidative stress‐related markers (MDA) and increase free radical scavenging enzymes (SOD and GPX) and the total antioxidant capacity (TAC) (Afsharpour et al., 2019; Jasprica et al., 2007). Table 3 presents the summary of the animal studies investigating the effects of propolis on oxidative stress.

**TABLE 3:** Summary of animal studies on the effects of propolis on oxidative stress

First author (year)	Country	Animal species	Propolis dosage	Duration (day)	Outcomes	Reference
Remirez et al. (1997)	Cuba	Rat	25, 50, and 100 mg/kg/day	–	↓ MDA in liver	Remirez et al. (1997)
Chopra et al. (1995)	India	Rat	50 and 100 mg/kg/day	–	↓ MDA	Chopra et al. (1995)
Rodriguez et al. (1996)	Cuba	Rat	10, 50, and 100 mg/kg/day	–	↓ MDA	Rodriguez et al. (1997)
Ilhan et al. (1999)	Turkey	Rabbit	10 μmol/kg/day	–	↓ MDA	Ilhan et al. (1999)
Ozyurt et al. (2001)	Turkey	Rat	10 μmol/kg/day	–	↓ MDA	Ozyurt et al. (2001)
Shinohara et al. (2002)	Japan	Rat	–	–	↓ LPO	Shinohara et al. (2002)
Shukla et al. (2004)	India	Rat	200 mg/kg/day	–	**↓** LPO ↑ Hepatic GSH level	Shukla et al. (2004)
Hu et al. (2005)	China	Rat	1 ml/100 g	56	↓ Fructose amine and MDA **↑** SOD	Hu et al. (2005)
Tan‐no et al. (2006)	Japan	Mice	At dilutions of 1:100 and 1:1000	–	↓ NO production	Tan‐no et al. (2006)
Sobocanec et al. (2006)	Croatia	Mice	100 mg/kg/day	–	↑ CAT and SOD ↓ TBARS	Sobocanec et al. (2006)
Eraslan et al. (2007)	Turkey	Rat	200 mg/kg/day	7 and 21	↔ Antioxidant enzymes and MDA levels	Eraslan et al. (2007)
Nirala and Bhadauria (2007)	India	Rat	100 and 200 mg/kg/day	–	GSH was restored by propolis treatment	Nirala and Bhadauria (2008)
Kismet et al. (2008)	Turkey	Rat	100 mg/kg/day	7	↓ Plasma and liver levels of MDA ↑ Liver GPX activities	Kismet et al. (2008)
Kanbur et al. (2008)	Turkey	Rat	100 mg/kg/day	28	↓ MDA levels ↑ SOD, CAT, and GPX	Kanbur et al. (2009)
Alyane et al. (2008)	Algérie	Rat	100 mg/kg/day	4	↓ MDA formation and production of superoxide anion	Alyane et al. (2008)
Yousef et al. (2009)	Egypt	Rat	50 mg /kg/day	70	↓ TBARS ↑ GSH, CAT, and GST	Yousef et al. (2009)
Zhao et al. (2009)	China	Mice	200 mg/kg/day	3	Propolis inhibited lipid peroxidation and oxidized ↑ GSH	Zhao et al. (2009)
Abo‐Salem et al. (2009)	Egypt	Rat	100, 200, and 300 mg/day	40	↓ MDA ↑ GSH and SOD activities	Abo‐Salem et al. (2009)
El‐Sayed et al. (2009)	Egypt	Rat	200 mg/kg/day	35	↓ MDA pancreatic content and serum NO ↑ Serum GSH and CAT activities ↑ Pancreatic SOD activities	El‐Sayed et al. (2009)
Khalil et al. (2010)	Egypt	Rat	Dietary propolis powder (0.1% and 0.2%)	42	↓ MDA ↑ GSH, SOD, and CAT activities	Khalil and El‐Sheikh (2010)
Nader et al. (2010)	Egypt	Rabbit	75 mg/kg/day	28	↓ TBARS ↑ GSH	Nader et al. (2010)
Seven et al. (2010)	Turkey	Broiler	1 g/kg/day	42	↓ MDA	Seven et al. (2010)
Zhu et al. (2010)	China	Rat	100 mg/kg/day	56	↓ MDA and NOS ↑ SOD and GPX	Zhu et al. (2011)
Bhadauria (2011)	India	Rat	200 mg/kg/day	14	↓ TBARS ↑ CAT and GSH	Bhadauria and Medicine (2012)
Zhu et al. (2011)	China	Rat	100 mg/kg/day	56	↓ Blood and renal MDA	Zhu, Chen, et al. (2011)
Garoui et al. (2011)	Tunisia	Rat	1 g propolis/100 g diet	–	Propolis ↑ activity of antioxidant enzymes (GPX, CAT, and SOD) and the level of GSH in the kidney	Garoui et al. (2012)
Yonar et al. (2011)	Turkey	Rainbow trout	50 mg/kg/day	14	↓ MDA ↑ SOD, GPX, GSH, and CAT	Yonar et al. (2011)
Attia et al. (2012)	Egypt	Rat	50 mg/kg/day	70	↑ CAT, SOD, and GPX ↓ LPO	Attia et al. (2012)
Oršolić et al. (2012)	Croatia	Mice	50 mg/kg/day	7	↓ MDA content in liver and kidney	Oršolić et al. (2012)
Gulhan et al. (2012)	Turkey	Rainbow trout	10, 20, and 30 PPM	96 hr	↓ MDA	Fuat Gulhan et al. (2012)
Selamoglu‐Talas et al. (2013)	Turkey	Rat	200 mg/kg/day	5	↑ CAT activity ↓ MDA	Selamoglu‐Talas et al. (2013)
El‐Awady et al. (2013)	Egypt	Isolated rat aorta	400 μg/ml	3 hr	In vitro: ↑ SOD ↓ MDA	El‐Awady et al. (2014)
Newairy et al. (2013)	Egypt	Rat	50 mg/kg/day	28	↓ TBARS ↑ CAT, SOD, and GSH	Newairy and Abdou (2013)
Su et al. (2014)	Taiwan	Hepatic stellate cells of rat	200 and 400 mg/kg/day	28	↓ MDA	Su et al. (2014)
Abou‐Zeid et al. (2015)	Egypt	Chick	0, 125, 250, and 500 mg/kg/day	42	↓ MDA ↑ SOD, GPX, and CAT activity	Abou‐Zeid et al. (2015)
Hemieda et al. (2015)	Egypt	Rat	50 or 100 mg/kg/day	42	↓ MDA ↑ GSH, SOD, CAT, and TAC	Hemieda et al. (2015)
Sameni et al. (2015)	Iran	Rat	100 and 200 mg/kg/day	42	↓ MDA ↑ The activity of SOD and GPX	Sameni et al. (2016)
Al‐Hariri et al. (2015)	Saudi Arabia	Rat	0.3 g/kg/day	14	↓ TBARS	Al‐Hariri et al. (2016)
Elissa et al. (2015)	Egypt	Rat	0.6 g/kg/day	21	↓ MDA ↑ GSH	Elissa et al. (2015)
Kismet et al. (2017)	Turkey	Rat	200 mg/kg/day	14	↑ Total thiol ↓ MDA	Kismet et al. (2017)
Arslan et al. (2016)	Turkey	Japanese quail	0.5, 1, and 1.5 g/kg/day	8–42	↓ MDA	Sur Arslan and Tatlı Seven (2017)
Gul Baykalir et al. (2016)	Turkey	Rat	100 mg/kg/day	–	↓ MDA ↑ GSH and CAT level	Baykalir et al. (2018)
Bazmandegan et al. (2017)	Iran	Mice	100 and 200 mg/kg	24 and 48 hr	↑ SOD and GPX activity ↓ LPO	Bazmandegan et al. (2017)
Gong et al. (2017)	China	Mice	10 μmol/kg/day	28	↓ MDA and NO ↑ SOD and CAT activities and GSH	Gong et al. (2017)
El Rabey et al. (2017)	Saudi Arabia	Rat	20% w/w	28	↓ MDA ↑ CAT, SOD, and GST	El Rabey et al. (2017)
Alm‐Eldeen et al. (2017)	Egypt	Mice	0.2 mg/kg/day	14	↓ MDA ↑ GSH, CAT, and SOD	Alm‐Eldeen et al. (2017)
Rivera‐Yañez et al. (2018)	Mexico	Mice	300 mg/kg/day	15	↑ SOD, CAT, and GPX	Rivera‐Yañez et al. (2018)
Udo Nna et al. (2018)	Malaysia	Rat	300 mg/kg/day	28	↑ SOD, CAT, GPX, GSH, GST, and GSR ↓ MDA	Nna et al. (2018)
Aydin et al. (2018)	Turkey	Rabbit	200 mg/kg/day	28	↓ MDA ↑ GPX and CAT	Aydin et al. (2018)
Chen et al. (2018)	Taiwan	Rats	183.9 and 919.5 mg/kg/day	56	↑ SOD and GPX ↓ TBARS	Chen et al. (2018)
Abdel‐Rahman et al. (2019)	Egypt	Rat	50 and 100 mg/kg/day	–	↓ MDA ↑ SOD, CAT, GPX, and GSH	Abdel‐Rahman et al. (2020)
Shi et al. (2019)	China	Rat	200 mg/kg/day	84	↓ ROS ↓ RNS	Shi et al. (2019)

Abbreviations: ↑, increase; ↓, decrease; ↔, no effect; C, control; CAT, catalase; GPX, glutathione peroxidase; GSH, glutathione; GSR, glutathione reductase; GST, glutathione *S*‐transferases; LPO, lipid peroxidation; MDA, malondialdehyde; NO, nitric oxide; NOS, nitric oxide synthases; RNS, reactive nitrogen species; ROS, reactive oxygen species; SOD, superoxide dismutase; T, treatment; TAC, total antioxidant capacity; TBARS, thiobarbituric acid reactive substances.

In a clinical trial conducted by Mujica et al. (2017), propolis supplementation (15 drops twice a day) was reported to decrease thiobarbituric acid reactive substances and increase plasma glutathione (GSH). However, other studies have not confirmed the significant effects of propolis on improving oxidative stress (Gao et al., 2018; Zhao et al., 2016). According to Ebeid et al. (2016), consuming propolis capsules (1200 mg/day) for 10 days before radiotherapy significantly decreased MDA and increased TAC during and 10 days after the treatment. Furthermore, Hesami et al. conducted a double‐blind, randomized‐controlled trial on 62 patients with type II diabetes, reporting that propolis supplementation (500 mg/kg; thrice a day) improved the antioxidant defense mechanisms after 8 weeks by increasing the CAT activity (Hesami et al., 2019). The overexpression of mitochondrial catalase has been shown to diminish the incidence of arteriosclerosis in mice while exerting protective effects against cardiovascular dysfunction and injuries in human subjects (Lei et al., 2016; Tehrani & Moosavi‐Movahedi, 2018). In this regard, Gao et al. reported that after 18 weeks of consuming Chinese propolis, a significant increase was observed in the serum GSH, flavonoids, and polyphenols of patients with type II diabetes (Gao et al., 2018). Recently Soleimani et al. also observed that the administration of propolis (450 mg/kg twice daily) significantly increased the levels of GSH and TAC and decreased total oxidant status (TOS) and MDA after 4 weeks of intervention (Soleimani et al., 2021).

According to the literature, the main antioxidant mechanisms of propolis polyphenols may be associated with their scavenging effects on ROS, while nitrogen species and chelating metal ions may also be involved in the production of free radicals, reduction of xanthine oxidase reaction, and synergistic effects with other antioxidants (Kurek‐Górecka et al., 2013; Mujica et al., 2017). It is known that phenolic compounds, such as those found in propolis, act as antioxidants by interrupting the chain reaction of lipids (Torel et al., 1986), blocking chemiluminescence processes (Georgetti et al., 2003), and scavenging ROS (Bors et al., 1990). The antioxidant and reductive capacity of propolis against ROS could be attributed to two main mechanisms, namely the capacity of CAPE in activating NrF2 transcription factor (a regulatory protein associated with antioxidant protection and improvement in antioxidant enzymes) and the phenolic acid and flavonoid contents of propolis (CAPE, quercetin, apigenin, *p*‐coumaric acid, cinnamic acid, and *p*‐vanillin), which neutralize free radicals and oxidant compounds (Ichikawa et al., 2002; Lee et al., 2010). Moreover, propolis has been shown to significantly enhance vitamin C levels in the plasma, kidney, stomach, small intestine, and colon (Seven et al., 2010). Propolis could be absorbed through the bloodstream and act as a hydrophilic antioxidant in the absorption of vitamin C (Seven et al., 2010). Figure 1 represents the hypothetical mechanism of the effects of propolis on the reduction of inflammation and oxidative stress.

### 2.4 Cardioprotective effects of propolis in RA

Rheumatoid arthritis is an inflammatory disease associated with the increased risk of cardiovascular mortality and morbidity (Aviña‐Zubieta et al., 2008; Gonzalez‐Gay et al., 2005). However, the exact mechanism of the elevated risk of cardiovascular diseases (CVDs) in RA patients should be further explored. The increased risk of CVD in RA patients may be due to dyslipidemia. Several observational studies have demonstrated that RA is associated with negative effects on lipid profile (Boers et al., 2003; Park et al., 1999, 2002). Dyslipidemia causes atherosclerosis and CVD (Nelson, 2013; Tietge, 2014), whereas reduced serum cholesterol leads to a significantly lower risk of CVD (González‐Gay & González‐Juanatey, 2014; Stamler et al., 2000). Furthermore, inflammation in RA patients plays a pivotal role in disease progression (González‐Gay & González‐Juanatey, 2014). Scientific evidence suggests that chronic inflammation in patients with RA is associated with a higher risk of CVD (Gonzalez‐Gay et al., 2007; Gonzalez‐Gay et al., 2005). Inflammation causes oxidative changes, which influence the structure of high‐density lipoprotein (HDL) and decrease apolipoprotein‐A1 in RA patients (Charles‐Schoeman et al., 2009). In addition, the levels of the antioxidant enzyme associated with HDL (paraoxonase‐1) have been reported to be lower in patients with RA compared to healthy controls (Charles‐Schoeman et al., 2012).

The cardioprotective effects of propolis have been confirmed in several studies (Ahmed et al., 2017; Alyane et al., 2008; Daleprane & Abdalla, 2013). The in vitro and in vivo studies in this regard have also clarified the molecular mechanisms of these effects, some of which include the improvement in glucose and lipid profiles; reduced activity of scavenger receptors, inflammatory cytokines, and oxidative stress; improvement in endothelial function; and prevention of platelet aggregation (Daleprane & Abdalla, 2013). Moreover, numerous findings have indicated that polyphenols reduce CVD risk and inhibit the formation of atherosclerosis plaques (Gorinstein et al., 2011; Grassi et al., 2008; Norata et al., 2007). Therefore, propolis is considered as an abundant source of polyphenols with a potential role in preventing cardiovascular events.

Propolis has beneficial effects on the regulation of lipid and lipoprotein metabolism. Previous findings have indicated that propolis administration led to reducing liver cholesterol and triglyceride levels and hepatic triglyceride synthesis in rats (Daleprane et al., 2012; Hu et al., 2005). Moreover, treatment with Brazilian propolis in low‐density lipoprotein (LDL) receptor knockout mice decreased the levels of triacylglycerol (TAG), total cholesterol (TC), and non‐HDL‐C (Daleprane et al., 2012). The mice receiving propolis treatment also experienced a significant reduction in TAG and TC, as well as increased HDL‐C, compared to the untreated mice. On the same note, Turkish propolis has been reported to prevent alcohol‐induced acute liver injury and lipid deposition, exerting positive effects on the lipid profile. Notably, in the mice receiving propolis treatment and alcohol, HDL levels have been reported to be high, and LDL was observed to be lower compared to the mice receiving alcohol only (Kolankaya et al., 2002).

In other studies, propolis has shown favorable effects on the HDL and LDL levels of rats (Hu et al., 2005). For instance, administration of propolis in diabetic rats led to decreased levels of TC, LDL‐C, very‐low‐density lipoprotein (VLDL), and TAG. These findings highlight the role of propolis in the regulation of lipid metabolism, as well as its contribution to the status of lipid abnormalities (Hu et al., 2005). Daleprane et al. hypothesized that propolis might prevent atherosclerosis. On the contrary, treatment of LDL receptor gene (LDLr^−/−^) mice with the extracted polyphenols of propolis has been reported to decrease the area of atherosclerotic lesions (Daleprane et al., 2012) and prevent the development of atherosclerosis in the treated LDL r^−/−^ mice by improving the lipid profile and downregulating pro‐inflammatory cytokines, chemokines, and angiogenic factors. In the mentioned study, propolis administration also decreased the mRNA expression of several genes (MCP‐1, INF‐g, IL‐6, CD36, and TGF‐β), which play a key role in the atherosclerotic process (Daleprane et al., 2012).

Previous studies have demonstrated the beneficial effects of propolis on the lipid profile (Burdock, 1998; Castaldo & Capasso, 2002; Hu et al., 2005; Munstedt & Zymunt, 2001; Murata et al., 2004; Nader et al., 2010). For instance, Kolankaya et al. conducted an animal study and reported that the EEP at 200 mg/kg BW/day decreased LDL levels and increased HDL levels in rats (Kolankaya et al., 2002). Consistently, the results of another study indicated that the extracted polyphenols of red propolis significantly lowered TAG and TC and increased HDL‐C in the LDL r^−/−^ knockout mice (Daleprane et al., 2012). In a clinical trial conducted by Mujica et al., propolis supplementation for 90 days significantly increased HDL levels and decreased the systolic and diastolic blood pressure, thereby reducing the risk of CVDs (Mujica et al., 2017). In contrast, Samadi et al. indicated that supplementation with propolis (900 mg/day), in comparison with placebo, after 12 weeks had no significant effects on lipid profile (LDL, HDL, TC, TG, and VLDL) (Samadi et al., 2017). Also, Fukuda et al., in a randomized, controlled 8‐week trial, investigated the effect of Brazilian green propolis (226.8 mg/day). There were no significant effects of the propolis supplements on LDL, HDL, TC, and TG (Fukuda et al., 2015). Recently, Salehi‐Sahlabadi et al. (2020) in a systematic review and meta‐analysis of randomized‐controlled trials, indicated that propolis consumption is associated with a decrease in TG levels as well as an increase in HDL levels.

The proposed hypocholesterolemic mechanism of propolis is through the protein expression of the ATP‐binding cassette (ABC) transporters A1 and G1 (ABCA1 and ABCG1) (Gorinstein et al., 2011). Various types of propolis could increase *ABCA1* gene expression (Ichi et al., 2009; Koya‐Miyata et al., 2009), which in turn increases HDL and enhances the cholesterol efflux from the peripheral tissue (Chung et al., 2010; Daleprane et al., 2012; Nader et al., 2010). Therefore, propolis may improve the lipid profile by upregulating ABCA1 gene expression. In addition, the ethanol extract of Brazilian red propolis has been reported to increase the ABCA1 promoter activity in THP‐1 macrophages (Iio et al., 2012). Given that patients with RA have impaired ATP‐binding cassette G1‐mediated CEC due to the disease activity and its complications (Ronda et al., 2014), propolis supplementation may effectively decrease the disease symptoms, thereby decreasing the risk of CVDs. Simultaneously with the increase in the ABCA1 cassette, Brazilian red propolis could upregulate ApoA‐1, which is involved in the cholesterol efflux by macrophages. The effects of propolis on ABCA1 could be attributed to the activation of PPARγ and LXRα (Iio et al., 2012). Table 4 presents a summary of the animal studies regarding the effects of propolis on the lipid profile.

**TABLE 4:** Summary of animal studies on effects of propolis on lipid profile

First author (year)	Country	Animal species	Propolis dosage	Duration (day)	Outcomes	Reference
Kolankaya et al. (2002)	Turkey	Rat	200 mg/kg/day	15	↓ TC and TG ↑HDL‐C	Kolankaya et al. (2002)
Hu et al. (2005)	China	Rat	1 ml/100 g	56	↓ TC, TG, LDL‐C, and VLDL‐C ↑ HDL‐C	Hu et al. (2005)
Abo‐Salem et al. (2009)	Egypt	Rat	100, 200, and 300 mg/kg/day	40	↓ Cholesterol, TG, LDL‐C, and VLDL‐C ↑ HDL‐C	Abo‐Salem et al. (2009)
Nader et al. (2010)	Egypt	Rabbit	75 mg/kg/day	28	↓ TC, TG, and LDL‐C ↑ HDL‐C	Nader et al. (2010)
Zhu et al. (2011)	China	Rat	10‐mg propolis per 100 g/kg/day	56	↓ TC	Zhu, Li, et al. (2011)
Bhadauria (2011)	India	Rat	200 mg/kg	14	↓ TG and TC	Bhadauria (2012)
Li et al. (2012)	China	Rat	50, 100, and 200 mg/kg/day	70	↓ TG ↔ TC, HDL‐C, and LDL‐C	Li et al. (2012)
Fang et al. (2013)	China	Mice	160 mg/kg/day	98	↓ TC, TG, and non‐HDL‐C	Fang et al. (2013)
Newairy et al. (2013)	Egypt	Rat	50 mg/kg/day	28	Normalized the TC, TG, and LDL‐C ↑ HDL‐C	Newairy and Abdou (2013)
Alqayim (2015)	Iraq	Rabbit	50 mg/kg/day	60	↓ TC, TAG, and LDL‐C ↑ HDL‐C	Alqayim (2015)
Al Ghamdi et al. (2015)	Saudi Arabia	Mice	100 mg/kg/day	28	↓ LDL‐C ↑ HDL‐C	Al Ghamdi et al. (2015)
Elissa et al. (2015)	Egypt	Rat	0.6 g/kg/day	21	↓ TC, LDL‐C, and TG ↑ HDL‐C	Elissa et al. (2015)
Albokhadaim (2015)	Saudi Arabia	Rat	1% and 2% of cholesterol diet	14	↓ TG and TC	Albokhadaim (2015)
Kismet et al. (2017)	Turkey	Rat	200 mg/kg/day	14	↓ TC, non‐HDL‐C, and TG	Kemal Kismet et al. (2017)
Gong et al. (2017)	China	Mice	10 μmol/kg/day	28	↓ TC, TG, and LDL‐C ↑ HDL‐C	Gong et al. (2017)
Chen et al. (2018)	Taiwan	Rat	183.9 and 919.5 mg/kg/day	56	↓ TC, LDL‐C, and TG ↑ HDL‐C	Chen et al. (2018)
Ibrahima et al. (2019)	Egypt	Rat	100 mg/kg/day	28	↓ TC, LDL‐C, and TG ↑ HDL‐C	Ibrahim et al. (2019)

Abbreviations: ↑, increase; ↓, decrease; ↔, no effect; C, control; HDL, high‐density lipoprotein; LDL, low‐density lipoprotein; T, treatment; TC, total cholesterol; TG, triglyceride.

In vitro and in vivo data have proposed that the positive properties of propolis, which have an effect on the lipid profile, could be involved in the atheroprotective effects of this compound. Furthermore, scientific reports suggest that the polyphenols extracted from propolis may be effective in the prevention of atherosclerosis (Salehi‐Sahlabadi et al., 2020; Silva et al., 2011).

Nitric oxide is an endothelium‐derived relaxing factor with vasodilatory and antiaggregative properties, which protects the blood vessels at low concentrations. However, the excessive NO produced by inflammatory cells may react with other nitrogen and oxygen species and stimulate oxidative stress (Ali et al., 2014). Several studies have reported increased NO levels in the serum of patients with RA (Ali et al., 2014; Ersoy et al., 2002; Mahmoud & Ismail, 2011). According to an animal study, propolis intake in diabetic mice resulted in the reduction of NO and NOS levels (Hu et al., 2005). Propolis reduces NO levels by decreasing NOS activity, thereby protecting the endothelial cells of the blood vessels. Furthermore, the EEP could prevent NO production by reducing iNOS expression in Raw 264.7 macrophages and inhibiting the catalytic activity of iNOS. On the contrary, excessive NO production is involved in the cardiovascular inflammatory process, and propolis may affect the regulation of NO levels through its anti‐inflammatory activities.

### 2.5 Strengths and limitations

This review study aimed to assess the effects of propolis on inflammation, oxidative stress, and cardiometabolic indices in RA patients. The main limitation of our study was the heterogeneity of the reported data in the reviewed studies, and quality assessment of the studies might have led to more accurate results for the generalization of the data. To the best of our knowledge, this is the first review study that has gathered an in‐depth scientific demonstration of the possible effects of propolis on patients with RA.

## 3 CONCLUSION

The present study suggested that propolis may have beneficial effects on oxidative stress biomarkers and inflammation process in RA patients due to its potent antioxidant and polyphenolic properties. Further studies particularly clinical trials must be conducted to demonstrate the definitive effects of propolis on multiple aspects of RA disease.

## CONFLICTS OF INTEREST

The authors also declare that they have no conflict of interest.

## ETHICAL APPROVAL

No ethical approval was required, as this is a review article with no original research data.

## INFORMED CONSENT

There were no study participants in this review article, and informed consent was not required.

## Data Availability

All the data used in this study can be made available on reasonable request.
